# Short chain fatty acids ameliorate immune-mediated uveitis partially by altering migration of lymphocytes from the intestine

**DOI:** 10.1038/s41598-017-12163-3

**Published:** 2017-09-18

**Authors:** Yukiko K. Nakamura, Cathleen Janowitz, Christina Metea, Mark Asquith, Lisa Karstens, James T. Rosenbaum, Phoebe Lin

**Affiliations:** 1 Casey Eye Institute, Oregon Health and Science University, Portland, Oregon, United States; 2 Division of Arthritis and Rheumatic Diseases, Department of Medicine, Oregon Health and Science University, Portland, Oregon, United States; 3 Devers Eye Institute, Portland, Oregon, United States; 4 Department of Medical Informatics and Clinical Epidemiology, Oregon Health & Science University, Portland, Oregon, United States

## Abstract

Short chain fatty acids (SCFA) are metabolites of intestinal bacteria resulting from fermentation of dietary fiber. SCFA are protective in various animal models of inflammatory disease. We investigated the effects of exogenous administration of SFCAs, particularly propionate, on uveitis using an inducible model of experimental autoimmune uveitis (EAU). Oral SCFA administration attenuated uveitis severity in a mouse strain-dependent manner through regulatory T cell induction among lymphocytes in the intestinal lamina propria (LPL) and cervical lymph nodes (CLN). SCFA also suppressed effector T cell induction in the CLN and mesenteric lymph nodes (MLN). Alterations in intestinal morphology and gene expression demonstrated in the EAU model prior to the onset of uveitis were blunted by oral SCFA administration. Using a Kaede transgenic mouse, we demonstrated enhanced leukocyte trafficking between the intestine and the eye in EAU. Propionate suppressed T effector cell migration between the intestine and the spleen in EAU Kaede mice. In conclusion, our findings support exogenous administration of SCFAs as a potential treatment strategy for uveitis through the stabilization of subclinical intestinal alterations that occur in inflammatory diseases including uveitis, as well as prevention of trafficking of leukocytes between the gastrointestinal tract and extra-intestinal tissues.

## Introduction

Immune-mediated uveitis is a heterogeneous group of disorders causing inflammation within the eye, and accounting for about 10% of blindness^1^. Alterations in the intestinal microbiome are strongly linked to the pathogenesis of various immune-mediated diseases including rheumatoid arthritis, inflammatory bowel disease, type 1 diabetes, multiple sclerosis, and ankylosing spondylitis^2,3^. We previously reported that oral antibiotic-mediated alteration in the intestinal microbiome influences the severity of uveitis in an inducible experimental autoimmune uveitis (EAU) model^4^. EAU is a T-cell-mediated autoimmune disease model induced by immunization with uveitogenic retinal antigens or by passive transfer of T cells against retinal antigens^5,6^.

Intestinal bacteria are sometimes considered a hidden metabolic organ, since they play a significant role in human nutrition and metabolism^7^. Intestinal bacteria consist of 100 trillion bacteria with up to 1000 species and 100-fold more genes than those in the human genome. Anaerobic bacteria such as *Firmicutes* and *Bacteroidetes* are the most abundant bacterial phyla in the intestine of human adults, and consist of over 90% of the intestinal commensal community^8^.

Recent studies have shown that anaerobic bacteria, such as *Firmicutes*, produce relatively high levels of short chain fatty acids (SCFAs) as a metabolite of fermentation of dietary fiber. These SCFAs are protective in immune-mediated diseases, such as multiple sclerosis^9^, colitis^10,11^, atopic disease^12^, and graft-versus-host disease^13^ partially through the expansion of regulatory T cells in the intestine, as well as through protective effects on the integrity of the intestinal epithelial barrier^14^. The most abundant SCFAs produced in the intestine include acetate (C2), propionate (C3), butyrate (C4), and valerate (C5)^15,16^.

Current therapeutic options for immune-mediated uveitis typically target inflammatory pathways and have immunosuppressant effects, rather than targeting immune regulation that contributes to immune homeostasis. Immunosuppressants, such as systemic or local corticosteroids, are the most commonly used treatment for immune-mediated uveitis, but these agents can cause serious, life-threatening systemic or blinding ocular side effects. Therefore, the development of less toxic therapeutic agents is of the utmost importance.

In an effort to investigate novel treatment strategies for immune-mediated uveitis that might target immune regulation, we investigated the effects of exogenous orally administered SCFAs on uveitis severity, testing the hypothesis that oral SCFAs might attenuate disease by altering T cell subsets and/or enhancing intestinal homeostasis.

## Results

### Exogenously administered oral SCFAs reduce the severity of EAU in a strain-dependent manner

**Figure 1:**
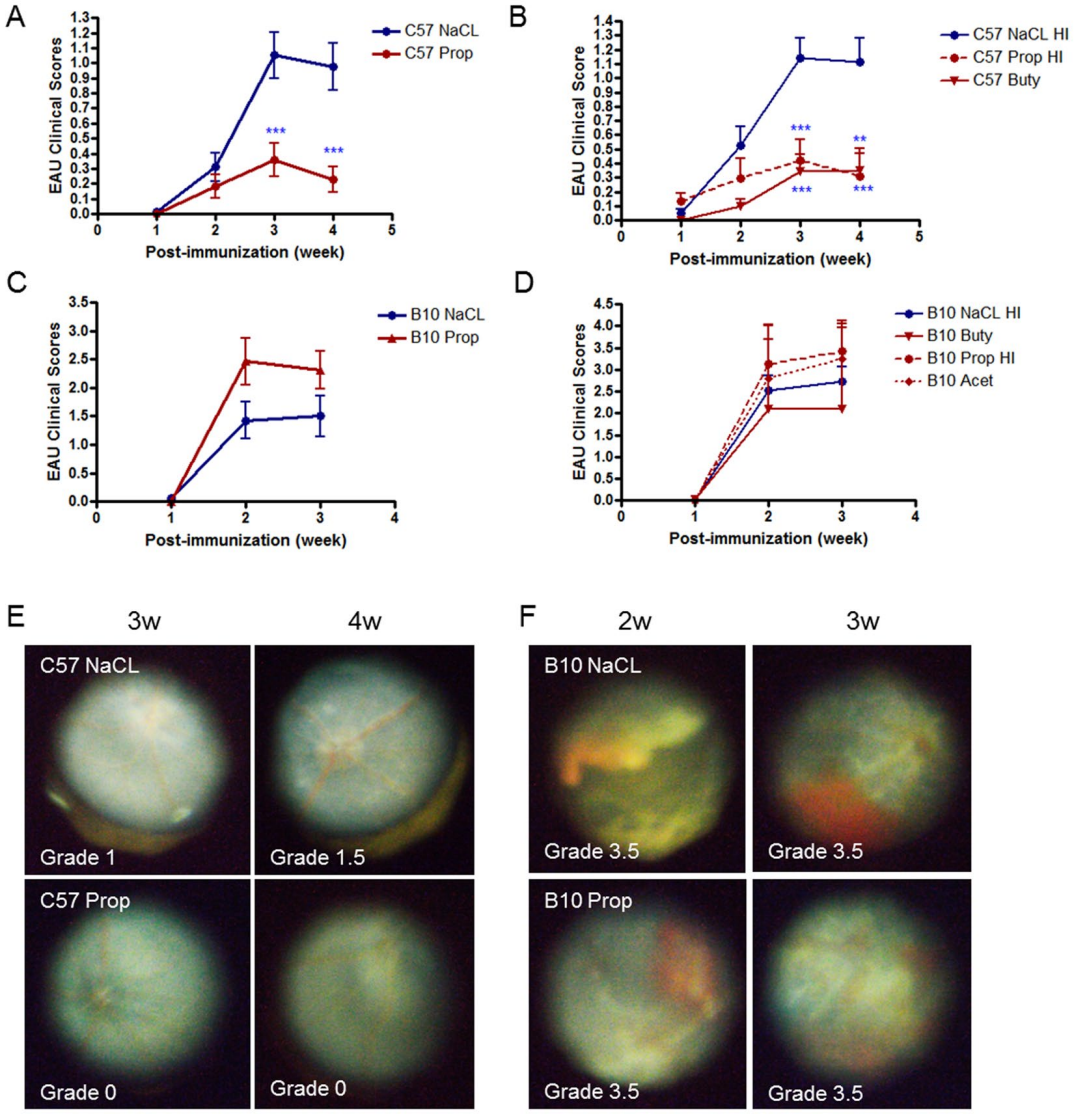
Orally administered SCFAs attenuate experimental autoimmune uveitis (EAU) in C57Bl/6J, but not B10.RIII mice. (**A**) EAU clinical scores in sodium propionate (Prop) vs sodium chloride (NaCl)-treated C57Bl/6J EAU mice. (**B**) EAU clinical scores in high dose Prop or butyrate (Buty) vs NaCl treated C57Bl/6J EAU mice. (**C**) EAU clinical scores in Prop vs NaCl-treated B10.RIII EAU mice. (**D**) EAU clinical scores in Prop, Buty, Acetate (Acet) vs NaCl-treated B10.RIII EAU mice. (**E**) Fundus photos of C57Bl/6J EAU mice treated with Prop vs NaCl at 3 and 4 weeks post-immunization. (**F**) Fundus photos of B10.RIII EAU mice treated Prop vs NaCl at 2 and 3 weeks post-immunization. n = 5–21 animals/treatment group; **p < 0.01, ***p < 0.0001; all values are expressed as mean ± SEM. NaCl: sodium chloride 150 mM; NaCl HI: sodium chloride 300 mM; Prop: 150 mM sodium propionate; Prop HI: 300 mM sodium propionate; Buty: 300 mM sodium butyrate; Acet: 300 mM sodium acetate.

To examine whether bacterial metabolite SCFAs affect the development of autoimmune uveitis, sodium propionate (Prop, 150 or 300 mM), sodium butyrate (Buty, 300 mM), or sodium acetate (Acet, 300 mM) was orally administered to C57Bl/6J or B10.RIII EAU mice in drinking water starting from day −21 prior to immunization (immunization day = day 0) until euthanasia. We used an inducible autoimmune uveitis model which differs in severity and time course between mouse strains, with C57BL/6J mice developing less severe uveitis over a longer period of time (peak at 3 weeks, convalescence at 4 weeks), and B10.RIII mice developing more severe uveitis over a shorter period of time (peak at 2 weeks, convalescence at 3 weeks)^6^. C57Bl/6J EAU mice treated with either 150–300 mM propionate (Prop or Prop HI) or 300 mM butyrate had significantly lower EAU clinical scores, starting at 3 weeks post-immunization, compared with EAU control animals given sodium-matched drinking water (NaCl 3w: 1.1 ± 0.7 vs. Prop 3w: 0.4 ± 0.5; NaCl HI 3w: 1.1 ± 0.6 vs. Prop HI 3w: 0.3 ± 0.4; NaCl HI 3w: 1.1 ± 0.6 vs. Buty 3w: 0.3 ± 0 0.4; p ≤ 0.001, n = 10 to 21) (Fig. 1A,B,E). However, B10.RIII EAU severity was unaffected by Prop, Buty, or Acet administration (Fig. 1C,D,F).

### Oral SCFAs alter T lymphocyte subsets

To investigate the mechanism of uveitis attenuation in C57Bl/6J EAU mice treated with oral SCFAs, we performed flow cytometry analysis to examine the frequency of regulatory T lymphocytes expressing FoxP3 and Helios in intra- and extra-intestinal lymphoid tissues: cecal and colonic lamina propria lymphocytes (LPL), cervical lymph nodes (CLN), mesenteric lymph nodes (MLN), and spleen (SPN). Because regulatory T lymphocytes play a key role in tolerance to self-antigens and prevention of autoimmunity, Tregs are one of the therapeutic targets for immune-mediated diseases^17^.

**Figure 2:**
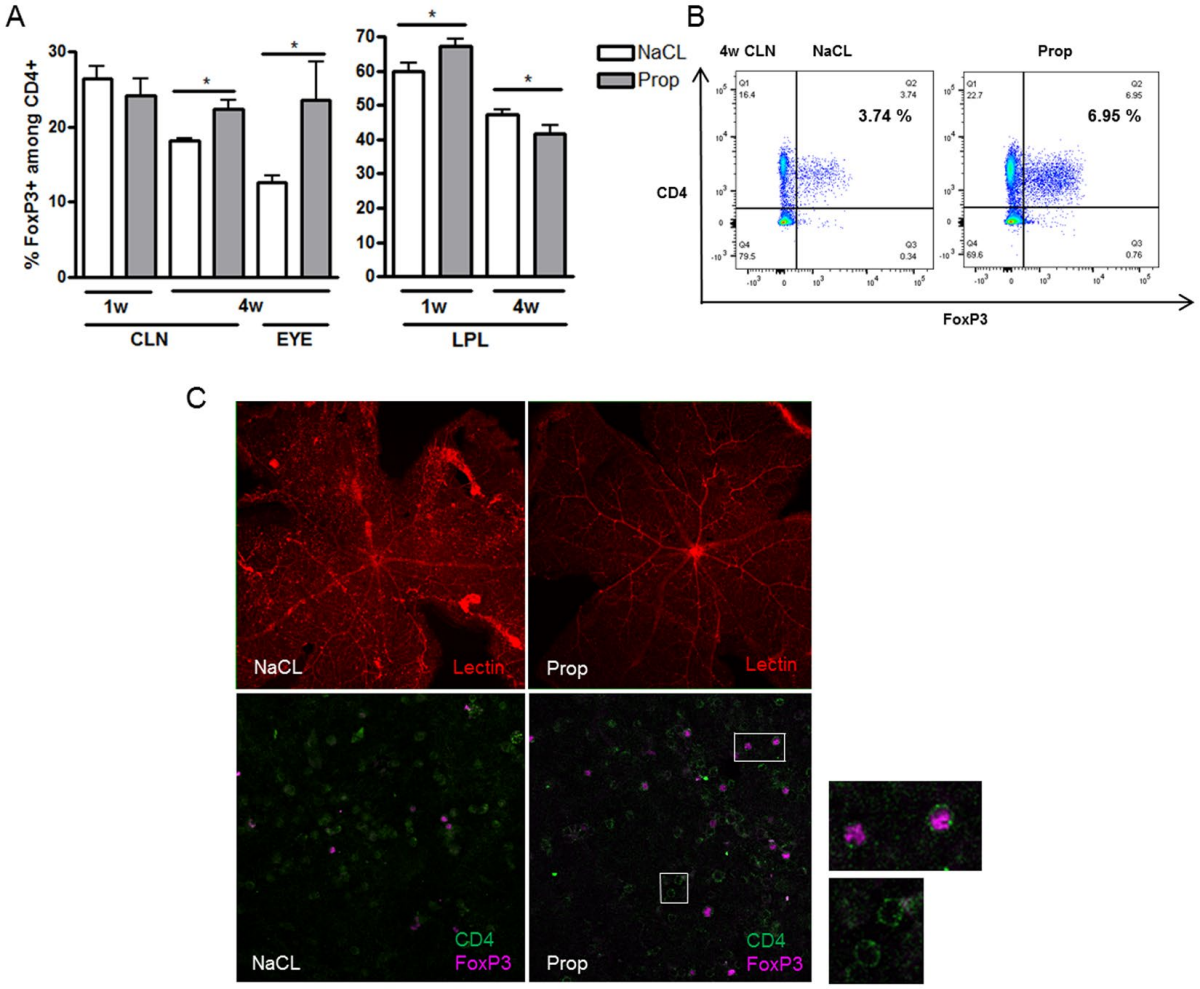
Propionate increases regulatory T cells in the intestinal lamina propria lymphocytes (LPL) early, and in the cervical lymph node (CLN)/eyes later, in EAU. (**A**) Flow cytometry shows increased Treg frequencies at 1 week (1w) in the LPL, and later at 4 weeks (4w) in the CLN and eyes (eyes quantitated by immunofluorescence). (**B**) Representative flow cytometry plots for (**A**). (**C**) Representative immunofluorescence retinal (eye) whole mounts from Prop vs. NaCl-treated C57Bl/6J EAU mice showing (top panels) lectin in red to demonstrate retinal vascular permeability and Tregs [FoxP3+ (magenta/pink), CD4+ (green)] in the bottom panel; high-magnification views to the right indicate localization of FoxP3 and CD4 staining. *p < 0.05, n = 5 animals per treatment group; representative of two separate experiments; all values are expressed as mean ± SEM.

Higher frequencies of FoxP3+, CD4+ Tregs were found in intestinal lymphoid tissues (cecal and colonic LPL) in SCFA-treated EAU mice than in the EAU control mice at 1 week post-immunization, prior to the onset of uveitis (NaCl 1w: 59.9 ± 5.6%vs. Prop 1w 65.3 ± 0.3%, p = 0.02), whereas Treg frequencies decreased in LPL by 4 weeks (NaCl 4w: 47.3 ± 4.7% vs. Prop 4w: 41.8 ± 7.0%, p = 0.03) (Fig. 2A). In contrast, Treg frequencies were increased by Prop treatment in CLN, the draining lymph node of the eye/head region, at 4 weeks (Fig. 2A,B). By 4 weeks, regulatory T cell proportions in the eyes were also increased by Prop treatment (quantified by immunofluorescence staining) (Treg in NaCl 12.6 ± 2.1% vs. Prop 23.6 ± 11.3%, p = 0.03), even though overall leukocyte infiltration and retinal vascular permeability by lectin staining in the retina was decreased by oral SCFA treatment (Fig. 2A,C). Despite testing several types and doses of SCFAs, these same treatments appeared to have minimal effect on Treg frequency in B10.RIII EAU mice (data not shown).

**Figure 3:**
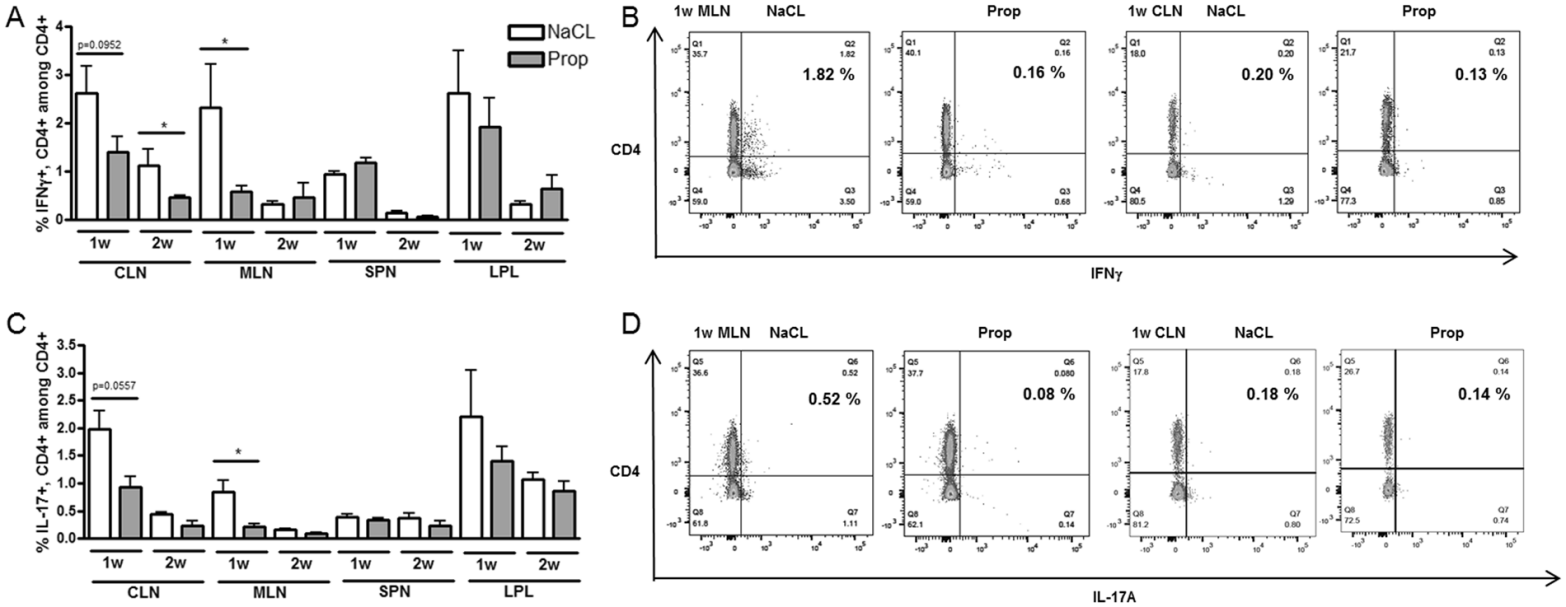
Propionate reduces Th1 and Th17 frequency in C57Bl/6J EAU. (**A**) Flow cytometry shows decreased Th1 prevalence in CLN and mesenteric lymph node (MLN) in propionate (Prop) vs sodium chloride (NaCl)-treated C57Bl/6J EAU mice. (**B**) Flow cytometric plots representative of data shown in (**A**). (**C**) Flow cytometry shows decreased Th17 prevalence in MLN and CLN in Prop vs NaCl-treated C57Bl/6J EAU mice. (**D**) Flow cytometric plots representative of data shown in (**C**). SPN: spleen, LPL: lamina propria lymphocytes; *p < 0.05, n = 5 per treatment group, representative of 2 separate experiments; all values are expressed as mean ± SEM.

In contrast to Tregs, there were decreased Th1 and Th17 frequencies early in the EAU time course (before the onset of uveitis) in CLN, MLN, and spleen of C57Bl/6J EAU mice treated with propionate (Th1: MLN NaCl 1w 2.3 ± 1.8% vs. Prop 1w 0.6 ± 0.2%, p = 0.03; CLN NaCl 1w 2.6 ± 1.3% vs. Prop 1w 1.4 ± 0.7%, p = 0.1, NaCl 2w 1.1 ± 0.8% vs. Prop 2w 0.5 ± 0.1%, p = 0.03) (Th17: MLN NaCl 1w 0.8 ± 0.4% vs. Prop 1w 0.2 ± 0.1%, p = 0.02; CLN NaCl 1w 2.0 ± 0.7% vs. Prop 1w 0.9 ± 0.4%, p = 0.06) (Fig. 3). This decrease in Th17 cells in MLN (the drainage lymph node for the intestinal tract), continued through 4 weeks, after the peak clinical score for EAU in C57BL/6J mice (Th17: MLN NaCl 4w 0.4 ± 0.1% vs. Prop 4w 0.3 ± 0.1%, p = 0.04; data not shown in figures), consistent with decreased IL-17 production shown by a Luminex assay (IL-17A: MLN NaCl 3w 14.87 ± 5.19 pg/ml vs. Prop 3w 5.35 ± 1.72 pg/ml, p = 0.008) (Supplemental Figure S1B) (IL-17A: MLN NaCl 4w 549.6 ± 33.3 pg/ml vs. Prop 4w 387.5 ± 14.74 pg/ml, p = 0.01; data not shown). A decrease in IFN_γ_ production by luminex assay was also found in both MLN and spleen at the peak of EAU clinical score (MLN NaCl 3w 108.6 ± 34.91 pg/ml vs. Prop 3w 40.54 ± 9.03 pg/ml, p = 0.008; SPN NaCl 3w 68.16 ± 14.84 pg/ml vs. Prop 3w 42.39 ± 12.85 pg/ml, p = 0.03) (Supplemental Figure S1A).

These results align with a recent study demonstrating that oral administration of SCFAs reduces disease severity by suppressing effector T cells and inducing Tregs in both multiple sclerosis and collagen-induced arthritis models^18^. Unlike its lack of effect on regulatory T cell populations in B10.RIII EAU, SCFAs suppressed Th17 cells in the MLN and Th1 cells in the cecal and colonic LPL (data not shown), but otherwise had either no effect or the opposite effect in other lymphoid tissues. Again, these T cell subset effects were not sufficient to suppress clinical ocular inflammation in B10.RIII mice.

### SCFA promotes intestinal homeostasis in EAU

Intestinal barrier dysfunction is characterized by alterations in intestinal morphology, gene expression, inflammation, and permeability. Nouri *et al*. reported that disruption of intestinal homeostasis preceded central nervous system (CNS) inflammation in experimental autoimmune encephalomyelitis (EAE), a model of CNS inflammation analogous to human multiple sclerosis, and similar in implementation to EAU^19^.

**Figure 4:**
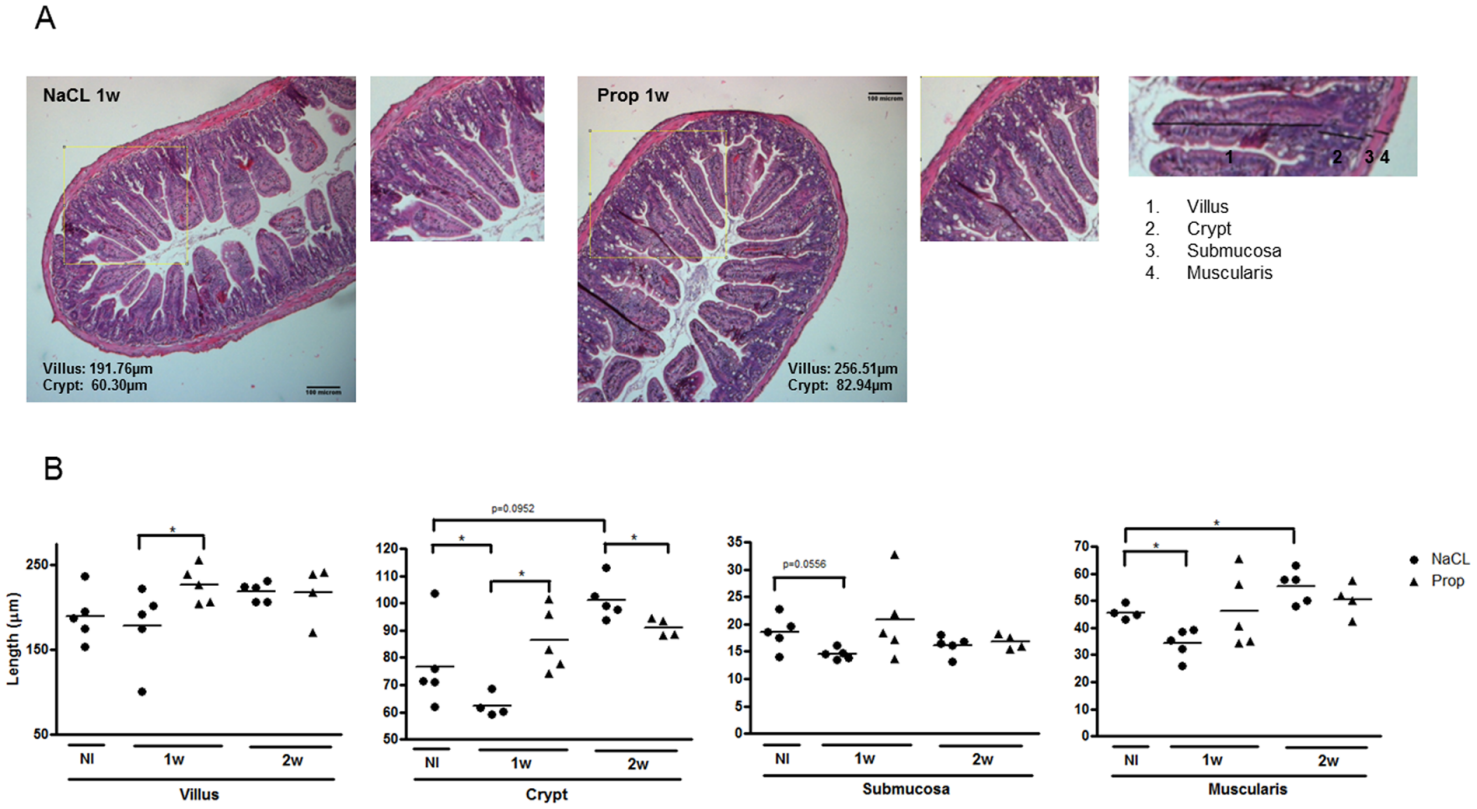
Orally administered SCFA maintains intestinal structural stability. (**A**) Hematoxylin and eosin stained sections of ileal tissue from sodium chloride (NaCl)-treated (left) vs propionate (Prop)- treated (right) C57Bl/6J EAU mice at 1 week post-immunization. Magnified inset on the far right demonstrates measurements for villus, crypt, submucosa, and muscularis. (**B**) Changes in villus, crypt, submucosa, and muscularis length (from left to right) associated with immunization and propionate treatment at 1 and 2 weeks post-immunization (1w, 2w). NI: non-immunized mice given sodium-matched water; *p < 0.05; n = 4–5 animals per treatment group, representative experiment out of two separate experiments; all values are expressed as mean ± SEM.

Intestinal architectural changes, such as crypt elongation and hyperplasia or villus flattening or atrophy, are signs of chronic intestinal inflammation in mice and humans^20,21^. In acute inflammation, for instance in certain viral infections, villus length and surface epithelium are reduced, whereas crypt length remains unchanged^21^. To investigate whether intestinal barrier dysfunction occurs in uveitogenesis, we examined intestinal morphology by measuring the length of four intestinal layers (the villus, crypt, submucosa, and muscularis) in the ileum in non-immunized (NI) vs. immunized (EAU) mice (Fig. 4A). At 1 week post-immunization (before the onset of uveitis), both intestinal crypt and muscularis length decreased significantly, compared to non-immunized mice given sodium-matched drinking water (crypt: NaCl EAU 1w 62.43 ± 4.24 μm vs. NI 76.77 ± 15.86 μm, p = 0.03; muscularis: NaCl EAU 1w 34.26 ± 5.41 μm vs. NI 45.71 ± 2.60 μm, p = 0.01) (Fig. 4B). Similarly, a non-significant decrease in submucosa length was observed in immunized vs. NI mice at 1 week (NaCl EAU 1w 14.52 ± 1.00 μm vs. NI 18.52 ± 3.17 µm, p = 0.06). On the other hand, at 2 weeks post-immunization, muscularis and crypt length increased in immunized vs. non-immunized mice (muscularis 2w: NaCl EAU 55.42 ± 6.16 µm vs. NI 34.26 ± 5.41 µm, p = 0.03; crypt 2w: NaCl 101.20 ± 7.36 µm vs. NI 76.77 ± 15.86 µm, p = 0.0952). In contrast to the ileal morphological changes early on (at 1 and 2 weeks post-immunization), there was no significant morphological change later in the time course of uveitis (at 3 and 4 weeks post-immunization, data not shown). It is important to note that clinically, no immunized animal was found to have diarrhea or weight changes, nor did they exhibit any other overt signs of gastrointestinal inflammation.

To investigate the effect of SCFA treatment on intestinal morphology, the above measurements were obtained in propionate-treated EAU mice. While propionate treatment increased crypt depth at 1 week post-immunization, it decreased crypt depth at 2 weeks, counteracting the changes seen at these time points caused by immunization (NaCl EAU 1w 62.43 ± 4.24 µm vs. Prop EAU 1w 86.42 ± 11.86 µm, p = 0.01, NaCl 2w 101.20 ± 7.36 µm vs. Prop EAU 2w 91.14 ± 3.30 µm, p = 0.03) (Fig. 4B). In other words, orally administered propionate appeared to stabilize changes in crypt depth that occurred in immunized mice before and after the onset of uveitis. Villus length was also increased by propionate at 1 week post-immunization (NaCl EAU 1w 178.30 ± 46.64 µm vs. Prop EAU 1w 226.50 ± 22.18 µm, p = 0.03).

**Figure 5:**
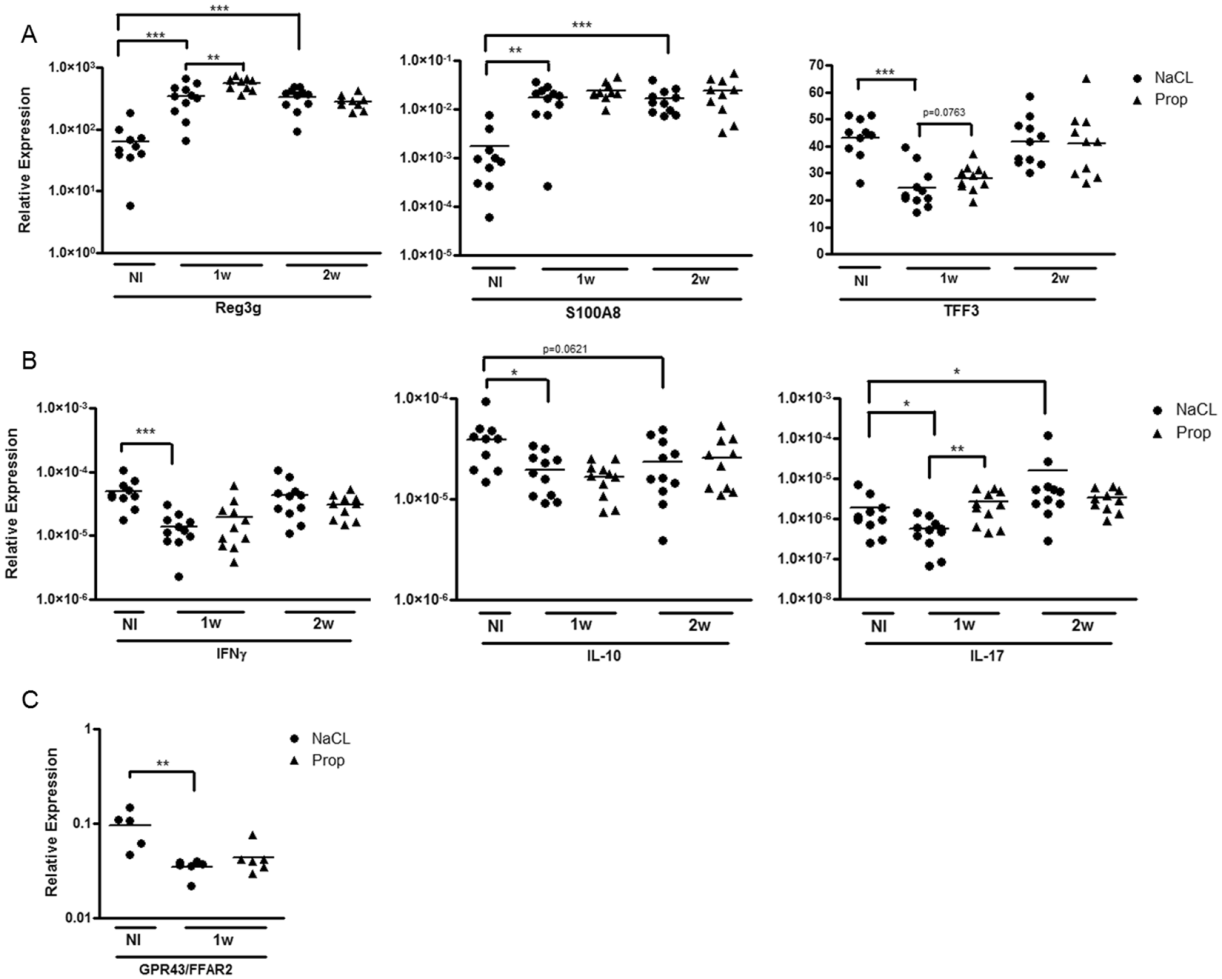
Effects of oral propionate on subclinical changes in ileal gene expression in uveitis. (**A**) Relative expression of anti-microbial peptides and trefoil factor 3 (TFF3) in immunized vs non-immunized (NI) mice, and the effect of oral propionate (Prop) administration. (**B**) Relative expression of cytokines in immunized vs NI mice, and the effect of Prop administration. (**C**) Relative expression of the G-protein coupled receptor GPR43 in non-immunized vs. immunized EAU mice with or without propionate treatment. NaCl: sodium chloride treated groups; IFN_γ_: interferon-gamma; IL-10: interleukin-10; IL-17: interleukin-17. *p < 0.05, **p < 0.01, ***p < 0.001; n = 10–11 animals per treatment group, all values are expressed as mean ± SEM.

Next, we assessed ileal gene expression involved in host defense, including anti-microbial peptides (AMPs) and cytokine production. Trefoil factor 3 (TFF3) is a peptide secreted by mucin-producing intestinal goblet cells, promoting mucosal protection, reconstitution, and repair^22^. TFF3-deficient mice are vulnerable to dextran sulfate sodium-induced colitis, whereas administering TFF3 promotes intestinal barrier function^23–25^. AMPs, including regenerating islet-derived protein 3 gamma (Reg3_γ_), S100A8 (calgranulin A), and lipocalin-2^26,27^ are secreted by Paneth cells located at the bottom of the crypt, and exert lytic, phagocytic, chemotactic activities, and are important in the clearance of endotoxins, such as bacterial lipopolysaccharide. At 1 week post-immunization (prior to the onset of uveitis), we found a significant reduction in TFF3; and increased S100A8 (NaCl EAU 1w 0.0179 ± 0.0105 vs. NI 0.00172 ± 0.00239, p = 0.002) and Reg3_γ_(NaCl EAU 1w 346.6 ± 179.1 vs. NI 64.33 ± 48.91, p = 0.0005) gene expression in immunized vs. non-immunized mice (Fig. 5A) thus suggesting subclinical alterations in intestinal homeostasis in the ileum prior to the onset of uveitis. At 2 weeks post-immunization, TFF3 returned closer to the non-immunized state, whereas the AMPs tested remained elevated. On the other hand, when inflammatory and regulatory cytokines were tested, the immunized state showed decreased IFN_γ_, IL-10, and IL-17 at 1 week (Fig. 5B) perhaps due to migration of cells producing these cytokines away from the gut.

We then looked at the effect of propionate treatment on these parameters. Whereas propionate treatment appeared to abrogate the effect on TFF3 gene expression, this was not statistically significant. Neither Reg3_γ_ nor S100A8 gene expression changes were reversed by propionate (Fig. 5A). Whereas reductions in IL-17 ileal gene expression induced by immunization were reversed by propionate treatment, neither IFN_γ_ nor IL-10 gene expression was affected (Fig. 5B). Overall, SCFA-treated EAU mice appear to be less affected by subclinical intestinal changes induced by immunization.

### Propionate reduces migration of effector T cells between the gut and the extraintestinal tissues

Kaede transgenic mice are a line of mice engineered to express the photo-convertible fluorescent Kaede protein in all cell types. These mice can thus be used to track the migration of leukocytes. Because the Kaede protein irreversibly changes in color from green to red through violet light exposure (405 nm), it allows us to monitor the movement of photo-converted red-labeled leukocytes from one location to another (Morton *et al*.^28^).

**Figure 6:**
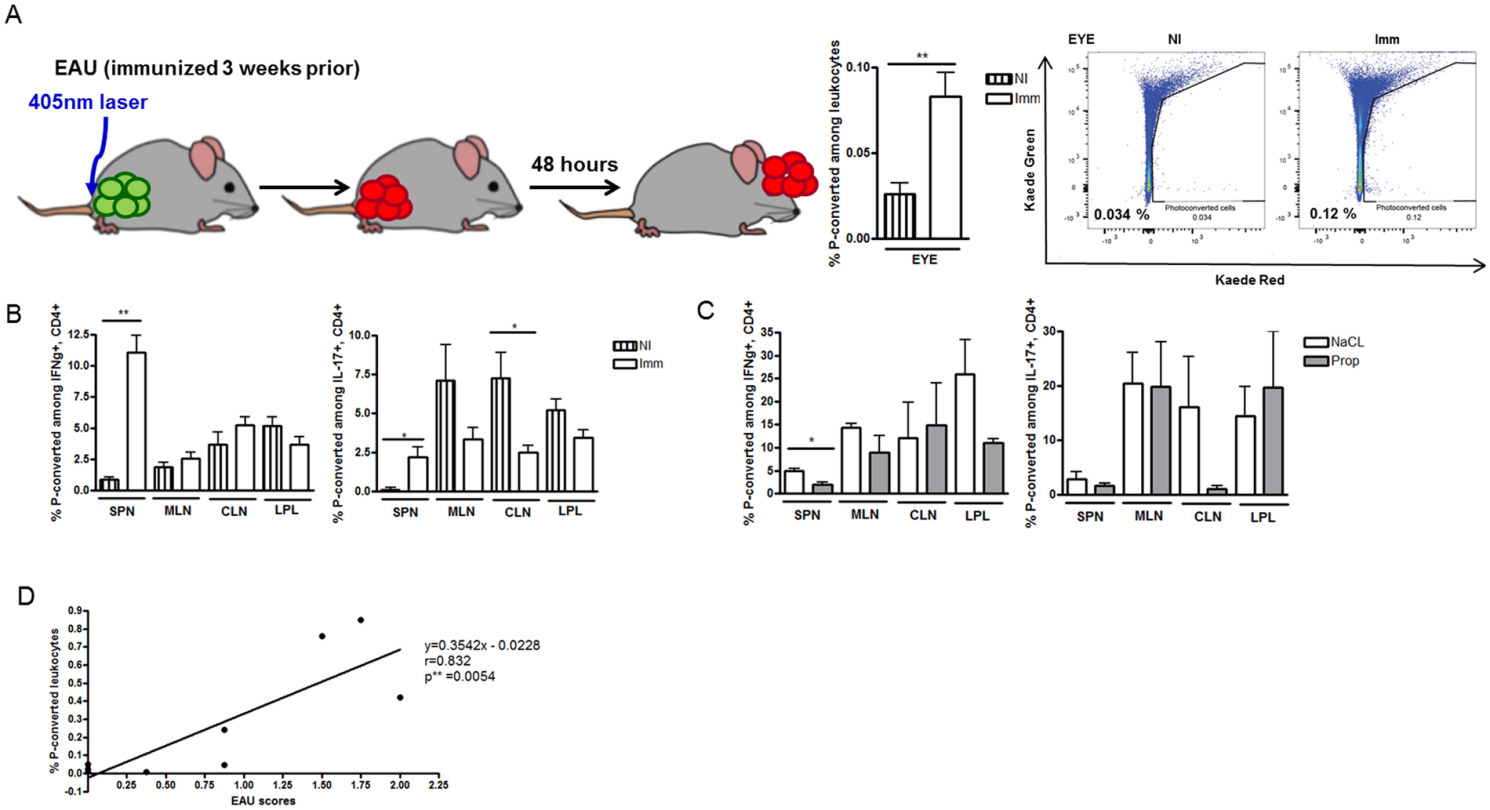
Propionate reduces migration of effector T cells between the gut and extra-intestinal tissues in experimental autoimmune uveitis (EAU). (**A**) Schematic of Kaede mouse photo-conversion (P-converted) in the colon with migration of leukocytes from the colon to the eye in EAU (graphs to the right). (**B**) EAU-induced migration of Th1 (IFN_γ_+, CD4+) (left) and Th17 (IL-17, CD4+) (right) cells from the colon to extra-intestinal lymphoid tissues. (**C**) Effects of oral propionate (Prop) treatment on Th1 and Th17 migration in EAU. (**D**) Pearson correlation test between EAU clinical scores and frequencies (%) of photo-converted leukocytes in the eye. SPN: spleen; MLN: mesenteric lymph node; CLN: cervical lymph node; LPL: lamina propria lymphocytes; NI: non-immunized; Imm: immunized (EAU mice); NaCl: sodium chloride control. *p < 0.05, **p < 0.01; n = 5–6 per treatment group; representative of 2 separate experiments; all values are expressed as mean ± SEM.

Using transgenic Kaede mice, we investigated leukocyte migration between the gastrointestinal tract and the eye using flow cytometry. For the first time, we demonstrated trafficking from the intestine to eye. Leukocytes labeled by photo-conversion in the colon were found in increased numbers in the inflamed eye with EAU (compared to non-immunized animals) (NI 0.03 ± 0.02% vs Imm 0.08 ± 0.04%; p < 0.01) (Fig. 6A). The severity of EAU and frequencies of photo-converted leukocytes in the eye were positively correlated (p = 0.005; Pearson’s r = 0.83; Fig. 6D). Although we detected relatively few leukocytes in the eye, Morton *et al*. also found that photo-converted leukocyte frequencies at as low as 0.14% in the intestinal Peyers Patches^28^. We also showed that both Th1 and Th17 cells migrated between the distal colon and the extra-intestinal lymphoid tissues at increased numbers when mice were immunized to induce uveitis (Th1: spleen NI 0.9 ± 0.3% vs Imm 11.1 ± 3.1%; p = 0.008) (Th17: spleen NI 0.3 × 10^−12^% vs. Imm 2.2%; p = 0.02) (Fig. 6B). We then used this model to investigate the effects of propionate administration. Oral propionate significantly reduced the migration of Th1 cells from the colon to the spleen in EAU, whereas it also reduced migration of Th17 cells although the latter was not statistically significant (Th1: NaCl 5.0 ± 1.2% vs. Prop 2.0 ± 0.8%; p = 0.02) (Th17: NaCl 2.9 ± 3.1% vs. Prop: 1.7 ± 0.7%; p = 0.79) (Fig. 6C). Other lymphoid tissues showed no difference with propionate administration. There was a trend towards decreased photo-converted leukocyte migration to the eye in propionate-treated animals, but no statistical comparison could be performed on pooled eyes. There were too few photo-converted effector T cells in eyes of propionate treated animals to perform statistical analyses on the latter.

Related to its effect on leukocyte migration, when we investigated chemokine production in the LPL, we found that propionate administration increased CXCL1 (otherwise known as KC) at 1 week post-immunization (NaCl 1w 269.4 ± 201.8 pg/ml vs. Prop 1w 588.9 ± 153.7 pg/ml, p = 0.03) (Supplemental Figure S1C). However, propionate significantly reduced CCL2 (monocyte chemoattractant protein-1) production in the LPL at peak EAU (NaCl 3w 25.16 ± 2.50 pg/ml vs. Prop 3w 13.79 ± 3.90 pg/ml, p = 0.03), thus suggesting that propionate can affect chemokine production in the intestinal tract. Other chemokines including CCL3, and CXCL9 were unaffected by propionate at either time point.

## Discussion

In summary, this study has demonstrated several novel findings. We discovered that exogenous administration of short chain fatty acids (intestinal bacterial metabolites of dietary fiber fermentation) attenuated uveitis in a mouse strain-dependent manner through induction of regulatory T cells in various tissues, as well as its suppressive impact on effector T cells. We showed that intestinal morphology and gene expression were altered in the EAU model prior to the onset of uveitis, and that some of these changes were blunted by oral SCFA administration. Finally, we, for the first time, also demonstrated the migration of leukocytes between the gastrointestinal tract and the eye in uveitis, and also showed that SCFAs reduced the migration of effector T cells between the intestine and the spleen during EAU. We and others have demonstrated the importance of the intestinal microbiome in the pathogenesis of uveitis^4,29,30^. Multiple mechanisms including the generation of SCFA potentially account for this effect. The demonstration that lymphocytes migrate from the intestine to the eye adds a new dimension to understanding the connection between the intestine and uveitis, especially in diseases such as Crohn’s disease, ulcerative colitis, ankylosing spondylitis, reactive arthritis, and psoriatic arthritis, since bowel and eye inflammation co-exist in each of these conditions^31,32^.

The mouse strain differences in SCFA-mediated attenuation of uveitis can be explained by several possible mechanisms. It is possible that the lack of regulatory T cell induction by SCFAs in B10.RIII mice resulted in a blunted suppressive effect on the effector arm of the immune system and thus resulted in a lack of clinical uveitis attenuation. This first explanation implies that Treg induction is a crucial portion of the SCFA effect in C57Bl/6J EAU mice. A second possible explanation is that SCFAs have less of a homeostatic effect on the gastrointestinal tract in B10.RIII mice (though not specifically tested) due to differences in intestinal microbial constituents. We have found that intestinal microbial constituents differ between the C57Bl/6J and B10.RIII mice (Supplemental Figure S2). For instance, it is possible that C57Bl/6J mice harbor more beneficial bacteria, including strains that produce SCFA or strains that can be manipulated by exogenous SCFA administration than the B10.RIII mice, thus resulting in strain-dependent amelioration with this treatment. Finally, the severity and phenotype of uveitis differ between the two strains of mice, thus implying that different pathways might need to be targeted for maximum therapeutic benefit in each mouse strain. These strain differences have implications for the application of oral SCFA administration in the treatment of human uveitis, which is a heterogeneous group of diseases with differing mechanisms, and might also imply that there may be individual variation in how one responds to this potential new therapy.

Previous studies have demonstrated that subclinical gastrointestinal inflammation can occur in the setting of extra-intestinal immune-mediated diseases other than uveitis. For instance, histopathologically-proven subclinical intestinal inflammation occurs in nearly 50% of subjects who have ankylosing spondylitis without any overt signs or symptoms of inflammatory bowel disease^33^. Furthermore, dysbiosis (or alteration of the intestinal microbiota towards the development of intestinal inflammation) occurs in other spondyloarthropathies; subjects who have a genetic risk allele, HLA-B27, which confers higher risk for spondyloarthropathy and uveitis, but who have neither disease overtly, also have dysbiosis^32^. In addition, dysbiosis and subclinical inflammation of the intestine has been implicated in multiple sclerosis^34^. In a mouse model of multiple sclerosis, EAE, which is analogous to EAU in implementation, Nouri *et al*. found structural and functional alterations in the intestinal tract before the onset of CNS inflammatory disease. They also demonstrated altered T cell subsets in the intestine prior to inflammation in the brain^19^. Given that uveitis can be associated with multiple sclerosis in patients, and that the two models are very similar, it is perhaps not surprising that we have found that alterations in intestinal gene expression and mucosal structure occur prior to the onset of uveitis in EAU.

Connecting the subclinical alterations in the intestine and the immune-phenotypic changes in extra-intestinal lymphoid tissues, as well as within the target tissues including the eye or the brain, has been elusive in prior studies. We propose, based on our experiments, that subclinical intestinal changes prior to the onset of uveitis create a permissive environment for migration of leukocytes from the gastrointestinal tract to extra-intestinal lymphoid tissues, and finally, the eye. Interventions such as oral SCFA treatment in our study, not only change the lymphocyte subsets in the intestine, but influence how they migrate to other tissues. Corroborating this migration theory is our data showing discordant IL-17 vs. Th17 levels found early on after immunization (1 week); propionate elevated IL-17 production in the ileum at a time when it reduced Th17 numbers in the MLN, suggesting that there may be propionate-induced retention of Th17 in the ileum prior to the onset of uveitis. Whether or not the observed intestinal structural and gene expression changes occurred directly as a result of SCFA administration versus indirectly as a result of changing inflammatory and regulatory cell populations is still unknown.

A number of studies reveal that SCFAs regulate gene expression, apoptosis, and host defense through activation of G protein coupled receptors (GPCRs) (*e.g*., GPR41 and GPR43) and through non-cell surface receptor mechanisms (*e.g*., the inhibition of histone deacetylases)^16,35–38^. There are multiple mechanisms by which SCFAs modulate inflammatory diseases. First, SFCAs may act as activators for GPCRs expressed on immune cells and/or intestinal epithelial cells (IPC). As suggested by Julia *et al*.^39^, migration of SCFA GPCR-stimulated tolerogenic dendritic cells to the draining MLN and circulation may contribute to the induction of Tregs and suppression of Th1/Th17 in distant tissues. We have shown suppression of Th1/Th17 in both the MLN and CLN early in the time course of our study, suggesting that similar effects are occurring. Serum propionate concentrations range from 3.8 to 5.4 μM^40^. As millimolar concentrations of SCFAs are required for GPR41 and GPR43 activation, SCFAs are likely to enable activation of these receptors exclusively in the intestine where there are much higher concentrations^41^. Therefore, it is plausible that orally administered SCFAs activate GPCR expressed on immune cells and epithelial cells found mainly in the intestinal tract in this study. A reduction in expression of the SCFA receptor, GPR43, in the ileum upon immunization might partially point towards a role for endogenous short chain fatty acids in the development of EAU, and it is possible that if the trend towards reversal of this reduction by oral administration of SCFA is true, this is may be one of the mechanisms for reduced EAU severity in propionate-treated animals.

Second, SCFAs may suppress T cell differentiation into Th1 and Th17 as well as induce Tregs through inhibition of histone deacetylase (HDAC), rather than through GPR41 or GPR43. Using an *in vitro* model, Park *et al*. demonstrated SCFA-mediated pro- and anti-inflammatory effects through HDAC inhibition. Since SCFAs can be absorbed by many cell types, SCFAs can bypass the cell-surface receptors to regulate cells without high expression of GPR41 or GPR43. The mTOR-S6K pathway effects on pro- and anti-inflammatory cytokines IFN_γ_, IL-17, and IL-10 has been implicated in this cell-surface receptor-independent SCFA effect. Others have found that orally administered SCFAs reduce pathogenic load by inducing AMP gene expression through inhibition of histone deacetylase activity^42,43^. Although neither GPCR-activation nor HDAC inhibition was investigated in depth in this study, it is plausible that both mechanisms are responsible for disease attenuation in EAU.

Treatment options have been suggested for intestinal microbiome-associated diseases, such as through oral supplementation of probiotics and fecal transplant. Significant portions of orally administered live bacteria fail to reach and colonize the large intestine due to exposure to stomach acid and bile salts in the upper GI tract^44^. In contrast, free fatty acids are not affected by digestive factors or metabolized until taken up by the colonocytes, liver, or peripheral tissues. In this regard, oral supplementation of SCFAs may be a relatively efficient and practical option among other potential strategies. However, the risks of long-term and/or high dose supplementation of SCFAs are yet unknown. For instance, a recent study demonstrated that T cell dysregulation and renal inflammation resulted from SCFA administration at higher than physiological levels^45^.

Our study (3 week pre-treatment of SCFAs) may support the potential of SCFA administration as a prevention strategy in chronic or recurrent uveitis, but the beneficial effects may be limited to certain populations depending on the underlying mechanism or severity of uveitis, as represented in our strain-specific findings. Other strategies, for instance, a high fiber diet to promote generation of closer to physiologic levels of intestinal SCFAs by the intestinal bacteria should be tested for a potentially safer long term alternative therapy. Furthermore, corroboration in clinical trials would be warranted to determine if exogenous SCFAs would be feasible, safe, and effective for uveitis patients.

In conclusion, we report a number of novel findings demonstrating subclinical intestinal dysfunction in uveitis leading to enhanced migration of leukocytes between the intestine and the eye. We also demonstrate the potential of an orally administered metabolite of commensal intestinal microbiota as a treatment strategy for uveitis through the stabilization of these subclinical alterations both inside and outside the gastrointestinal tract.

## Methods

### Mouse treatments (B10.RIII, C57BL/6J, Kaede/C57BL/6J)

Female 8- to 12-week-old C57BL/6J and B10.RIII mice were purchased from the Jackson Laboratory (Sacramento, CA, USA). Kaede mice on the C57BL/6 background were provided by Dr. Diane Mathis of Harvard University. Kaede mice can be provided from the RIKEN BRC through the National Bio-Resource Project of the MEXT, Japan^46^. Mice were maintained in ventilated cages under HEPA-filtered barrier conditions and fed gamma-irradiated standard chow, in accordance with the institutional policies for animal health and well-being at the Oregon Health & Science University. The experimental protocols were approved by and conducted following the guidelines of the Oregon Health & Science University Institutional Animal Care and Use Committee with strict ethical and humane treatment guidelines. Mice were treated with sodium propionate (Prop), sodium butyrate (Buty), or sodium acetate (Acet) (150 or 300 mM; Sigma-Aldrich, St. Louis, MO, USA) or sodium chloride (NaCl; sodium-equivalent to each SCFA treatment) as the control in drinking water, starting 3 weeks prior to immunization until euthanasia. These solutions were changed twice a week for the duration of the study.

### EAU induction

Female C57Bl/6J and Kaede transgenic (on C57Bl/6J background) mice were immunized subcutaneously into the base of the tail and each thigh with 200 µl total of the emulsion containing IRBP1–20 or IRBP651–670 (300–500 µg peptide/animal; AnaSpec, Fremont, CA, USA; GenScript, Piscataway, NJ, USA) made in incomplete Freund’s adjuvant (IFA; Sigma-Aldrich) with heat-inactivated *Mycobacterium tuberculosis* antigen (5 mg/ml; BD, Franklin Lakes, NJ, USA). In addition, pertussis toxin from *Bordetella pertussis* (1 µg dose/animal; Sigma-Aldrich) was injected subcutaneously once at the time of immunization per previously published reports^6^. Because the timing of EAU peak intraocular inflammation shifted from 3 weeks post-immunization to 16 days post-immunization depending on the IRBP supplier (AnaSpec vs. GenScript), time points were adjusted and equivalently expressed in our time course experiments. Female B10.RIII mice were immunized with 200 µl of the emulsion containing IRBP161–180 (15 µg peptide/animal; AnaSpec, Fremont, CA) made in IFA with the *Mycobacterium tuberculosis* antigen (2.5 mg/ml) as previously reported^4^. Clinical EAU scores were evaluated by fundus examination with a 90D lens (Volk, Mentor, OH, USA) and indirect ophthalmoscope (Keeler, Sacramento, CA, USA) weekly until euthanasia. Fundus photographical images were taken using an endoscope (Endoscopy Support Service, Brewster, NY, USA) and Nikon D3200 camera (Melville, NY, USA).

### Photo-conversion

Kaede transgenic mice were used to track the migration of immune cells. Fluorescently-labeled immune cells were photo-converted with violet light in one location and monitored for migration to another part of the body, as described by Morton *et al*.^28^. Kaede mice were anesthetized with 2.25% (v/v) isoflurane, while supplied 2.5 L/min of 100% oxygen. After flushing Kaede mice of colonic feces with PBS, the colon of each mouse was photo-converted (405 nm, power output 4.4 mW) by inserting a fiber cable attached to a custom-made violet-light source through the anus, retracting, and pausing at 2–2.5 mm increments for 1 minute (total 10 minutes). Forty-eight hours after photo-conversion, immune cells were isolated from the cecum and colon (LPL) or from other tissue sites as described below.

### Cell isolation

Single cell suspensions of lymphoid tissues: spleen [SPN; through RBC removal with ammonium-chloride-potassium (ACK) lysis buffer], cervical lymph node (CLN), and mesenteric lymph node (MLN), were obtained by processing through a 70 µm cell strainer. Lamina propria lymphocytes (LPL) were isolated from the cecum and colon. After removing their fecal contents, the cecum and colon were cut into 1 cm-long pieces, digested in RPMI media with 5 mM EDTA (37 °C, 30 minutes × 2), and subsequently with 0.1 mg/ml collagenase II (Sigma-Aldrich) and 0.1 mg/ml DNase I (Roche, San Francisco, CA, USA) at 37 °C for 45 minutes twice (total 90 minutes). Digested tissues were re-suspended in 30% Percoll (GE Healthcare, Pittsburgh, PA, USA) and then layered on a 40%/60% Percoll gradient. LPL were isolated from the 40%/60% Percoll interface after centrifugation at 740 × g for 20 minutes. Eyes were enucleated, the lens removed, and then minced. Two eyes (from one mouse) were pooled. The minced eye pieces were digested with 1 mg/ml collagenase D (Roche) and 15 µg/ml DNase I (Roche) at 37 °C for 40 minutes and processed through a 70 µm cell strainer.

### Flow cytometry

For regulatory T cell (Treg) frequency assessment, single cell suspensions processed were stained with an anti-mouse CD4-FITC antibody and Live/Dead dye-efluor780 (eBioScience, San Diego, CA, USA) after Fc blocking with rat anti-mouse CD16/CD32 Fc block (BD Pharmingen, San Jose, CA, USA). The suspensions were fixed with the FoxP3 Staining Buffer Kit (eBioScience) and then stained with antibodies against intracellular proteins: FoxP3-APC and Helios-efluor450 (eBioScience). For Th1 and Th17 cell detection, single cell suspensions were stimulated with 500 ng/ml phorbal 12-myristate 13-acetate (PMA, Sigma-Aldrich) and 2 μg/ml ionomycin (Sigma-Aldrich) in RPMI media (10% FBS, penicillin/streptomycin, β-mercaptoethanol) containing Golgi stop/monensin (BD BioSciences) at 37 °C for 3 hours. Subsequently, the stimulated cells were stained with an anti-mouse CD4-PE Cy7 (BD BioSciences) antibody and Live/Dead dye-efluor780, then fixed with intracellular cytokine detection kit (BD BioSciences) prior to staining with anti-mouse antibodies: IFN_γ_-FITC (BD BioSciences), IL-17A-AlexaFluor647 (BD Pharmingen), and TNFα-PE (eBioScience). For cells isolated from Kaede mice, anti-mouse antibodies CD4-PE Cy7, CD45 BV421 (BioLegend, San Diego, CA, USA) and IFN_γ_-APC-Cy7 (BioLegend) were used for Treg and/or Th1 and Th17 cell staining. Flow cytometric data were obtained through LSR Fortessa Cell Analyzer (BD BioSciences), and the data analyzed using FlowJo software (FlowJo LLC, Ashland, OR, USA).

### Luminex cytokine quantification

Supernatants were obtained from single cell suspensions of lymphoid tissues (SPN, MLN, CLN, LPL), which were stimulated with PMA and ionomycin at 37 °C for 3 hours in the presence of Golgi stop/monensin as described above, after centrifugation at 450 × g for 5 minutes. The supernatants were stored at −80 °C until the Luminex bead-based multiplex sandwich assay (Life Technologies). Cytokines and chemokines in the supernatants were captured by their specific antibodies with beads and streptavidin-conjugated fluorescent R-phycoerythrin, following the instructions provided for the assay. The proteins were detected and quantified through a dual laser flow-based analyzer Luminex 200 (Luminex Corp., Austin, TX, USA).

### Retinal whole mount preparation/confocal microscopy

Eyes were enucleated under anesthesia before euthanasia. Enucleated eyes were incubated in PBS for 10 minutes at RT and then in 4% paraformaldehyde/PBS for 15 minutes on ice. Retinas were removed carefully with Vanna’s scissors and fine forceps, washed with 0.1% Tween 20/Tris-buffered saline (TBS), and blocked with 2% normal goat serum/TBS for 2 hours. The retinas were stained with anti-mouse antibodies CD4-FITC and FoxP3-eFluor 570 (eBioScience) and Lectin-AlexaFluor647 (Life Technologies) overnight and then with DAPI (Invitrogen, Life Technologies) for 15 minutes, followed by mounting with Slowfade mounting media (Life Technologies). Confocal z-stacked images were obtained through an Olympus 7000 (Center Valley, PA, USA). The confocal images were converted to single channel split images before counting CD4-FITC+, FoxP3-eFluor 570+ or CD4-FITC+, FoxP3-eFluor 570- cells using ImageJ (NIH, Bethesda, MD, USA).

### Ileum structure measurement

Fresh ileum was obtained after euthanasia and cut into approximate 7 mm-long pieces. The ileal fragments were fixed with 10% neutral buffered formalin (NBF) overnight, and stored in 70% ethanol at 4 °C until tissue processing. These tissues were processed overnight using a tissue processor (Shandon Citadel 2000, Thermo Scientific), and paraffin-mounted sagittally. Ileal tissues were sectioned transversally at 6 μm, and then hematoxylin and eosin stained. Stained ileal tissues were imaged with Leica DM 5000B (Leica Microsystems Buffalo Grove, IL, USA). Image J was used to measure the length of the ileal villus, crypt, submucosa, and muscularis.

### Ileum gene expression

Fresh ileal tissue (7 mm-long fragment) was snap frozen with liquid nitrogen and stored at −80 °C until RNA isolation. Total RNA was extracted with TRIzol (Life Technologies), and cDNA synthesized at 37 °C using a cDNA synthesis kit (Thermo, Applied Biosystems). Gene expression of anti-microbial peptides (AMPs) was assessed using primer sets: Reg3A (Mm00441128_g1), S100A8 (Mm00496696_g1), Tff3 (Rn00564851_m1), FFAR2/Gpr43 (Mm01176528_g1), and HPRT (Mm01545399_m1) as the housekeeping gene, using the Maxima Probe/ROX qPCR Master Mix (Thermo Fisher Scientific, Grand Island, NY, USA). Gene expression of various cytokines was quantified using custom-made primer sets: IFN_γ_(forward: 5′-GCG TCA TTG AAT CAC ACC TG-3′, reverse: 5′-TGA GCT CAT TGA ATG CTT GG-3′)^47^, IL-10 (forward: 5′-GGT TGC CAA GCC TTA TCG GA-3′, reverse: 5′-ACC TGC TCC ACT GCC TTG CT-3′)^48^, IL-17A (forward: 5′-CTC AAA GCT CAG CGT GTC CAA ACA-3′, reverse: 5′-TAT CAG GGT CTT CAT TGC GGT GGA-3′) and GAPDH (forward: 5′-TCA ACA GCA ACT CCC ACT CTT CCA-3′, reverse: 5′-ACC CTG TTG CTG TAG CCG TAT TCA-3′)^49^, as the housekeeping gene, using SYBR Green reagents (Quanta Biosciences, Beverly, MA, USA) performed on a ViiA 7 Real-time PCR machine (Thermo Fisher Scientific). Relative expression of these target genes compared to internal controls (HPRT or GAPDH) was calculated.

### 16S rRNA gene sequence processing, taxonomic classification, and diversity analyses

Cecal contents were collected on euthanasia and stored at −20 °C until microbial DNA isolation. Microbial DNA was isolated from frozen cecal contents with a DNA isolation kit (Mo Bio Laboratories, Inc., Carlsbad, CA, USA). Amplification of the 16 S small subunit rRNA gene was performed using standard protocols of the Earth Microbiome Project (www.earthmicrobiome.org)^50^. The V4 region of the 16 S gene was targeted with universal primers 515 F/806RB and sequenced on the Illumina MiSeq. The sequences were processed using scripts implemented through the workflow package Quantitative Insights Into Microbial Ecology (QIIME) (v1.9.0)^51^. Individual sequence reads were assembled with FASTQ-join (ea-utils, version 1.1.2–686), with default settings. Operational taxonomic units (OTUs) were picked with open reference OTU-picking using uclust and the greengenes reference database (gg_13_8)^52^. Chimeric sequences were removed with the blast_fragments approach implemented in QIIME. Taxonomy was assigned to individual OTUs using uclust with a minimum of 90% similarity to consider a database match a hit and minimum consensus fraction of 0.51. Beta diversity was assessed with weighted UniFrac and visualized with Principal Coordinate Analyses (PCoA). Permutational multivariate analysis of variance (PERMANOVA) was performed to determine statistical significance between the two mouse strains’ intestinal microbiota using weighted UniFrac. To identify differentially abundant OTUs between mouse strains, we utilized LEfSe analysis developed by the Huttenhower lab (https://huttenhower.sph.harvard.edu/galaxy/) which uses a non-parametric factorial Kruskal-Wallis (KW) sum-rank test to detect features with significant differential abundance; biological significance was then determined using pairwise tests using the Wilcoxon rank-sum test. Finally, LEfSe uses Linear Discriminant Analysis to estimate the effect size of each differentially abundant feature^53^.

### Other statistical analysis

Grubb’s test was performed on each treatment group to identify outliers. A nonparametric Mann-Whitney U test was performed to compare treatment groups using GraphPad Prism 4 (GraphPad Software, Inc., La Jolla, CA, USA). Data are expressed as mean ± SEM, and p < 0.05 considered significant. Pearson correlation test was used to compare clinical EAU score with frequency of photoconverted cells in the eyes of Kaede mice.

### Data availability

All data generated or analyzed during this study are available from the corresponding author on reasonable request.

## Electronic supplementary material

Supplementary figures
