# Supplementary material for: Short chain fatty acids ameliorate immune-mediated uveitis partially by altering migration of lymphocytes from the intestine
Source: Sci Rep. 2017 Sep 18;7:11745. doi: 10.1038/s41598-017-12163-3 (PMC5603543; doi:10.1038/s41598-017-12163-3)
Supplement: Supplementary file 1 — Supplementary figures [file 41598_2017_12163_MOESM1_ESM.pdf]

**Supplementary figures**

## Title

Short chain fatty acids ameliorate immune-mediated uveitis partially by altering migration of lymphocytes from the intestine

## Authors

Yukiko K. Nakamura[1], Cathleen Janowitz[1], Christina Metea[1], Mark Asquith[2], Lisa Karstens[4], James T. Rosenbaum[1, 2, 3,] and Phoebe Lin[1]

[1]Casey Eye Institute, Oregon Health and Science University, Portland, Oregon, United States

[2]Division of Arthritis and Rheumatic Diseases, Department of Medicine, Oregon Health and Science University, Portland, Oregon, United States

[3]Devers Eye Institute, Portland, Oregon, United States

[4]Department of Medical Informatics and Clinical Epidemiology, Oregon Health & Science University

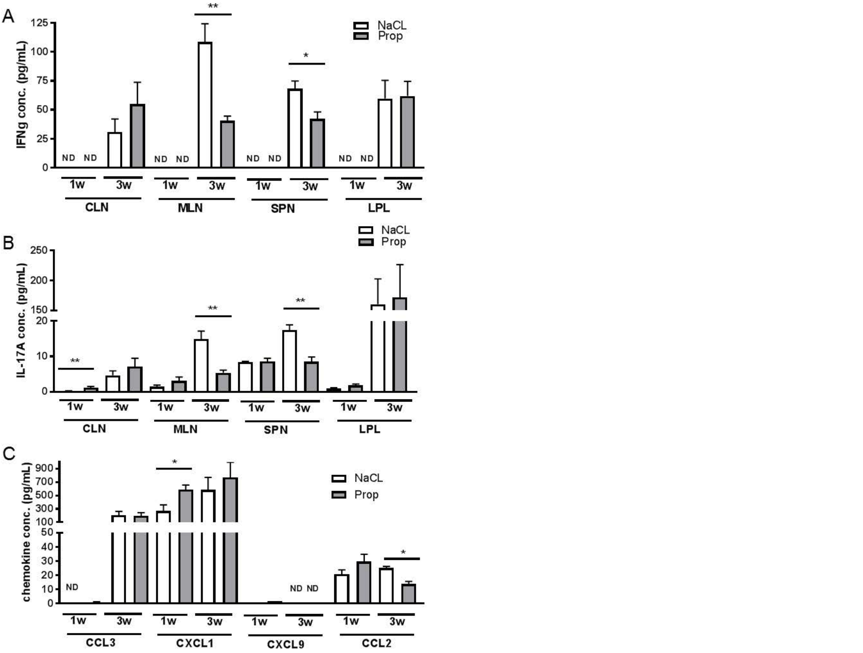


Figure S1. Luminex cytokine data showing interferon-gamma (A) and IL-17 (B) production in various lymphoid tissues in EAU with propionate treatment. (C) Chemokine production in lamina propria lymphocytes of EAU mice treated with propionate; CLN: cervical lymph node; MLN: mesenteric lymph node; SPN: spleen; LPL: lamina propria lymphocytes from the colon and cecum ; ND: non-detectable

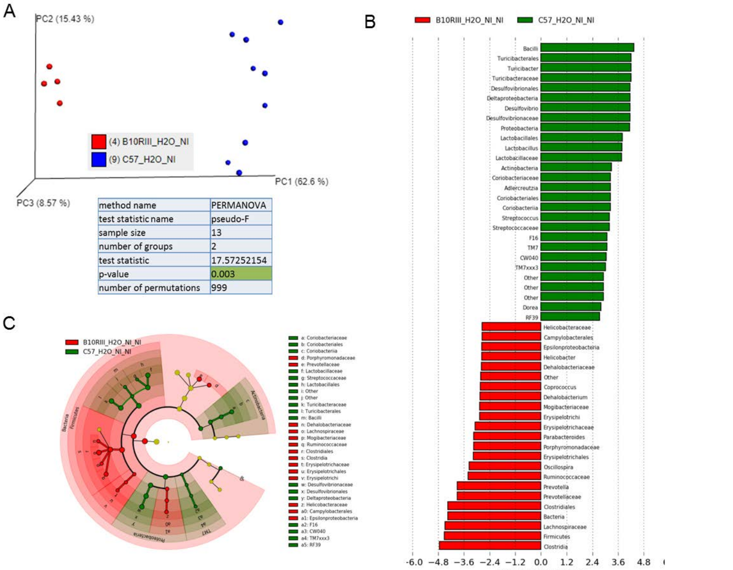


| | |
|---|---|
| method name | PERMANOVA |
| test statistic name | pseudo-F |
| sample size | 13 |
| number of groups | 2 |
| test statistic | 17.57252154 |
| p-value | 0.003 |
| number of permutations | 999 |

Figure S2. Mouse strain differences in the intestinal (cecal) microbiome.

(A) Principal coordinates analysis plot of 16S sequencing data from weighted unifrac analysis for beta diversity between C57Bl/6J and B10.RIII mice. All mice were healthy and non-immuinized. Permanova results are found in the table below the graph.

(B) Linear discriminant analysis effect size (LEfSe) results determining biologically significant differences in bacterial genera between C57 and B10.RIII mice

(C) Cladogram results showing bacterial strain differences between C57 and B10.RIII mice as determined by LEfSe analysis
